# Supplementary material for: Altered microRNA Transcriptome in Cultured Human Liver Cells upon Infection with Ebola Virus
Source: Int J Mol Sci. 2021 Apr 6;22(7):3792. doi: 10.3390/ijms22073792 (PMC8038836; doi:10.3390/ijms22073792)
Supplement: Supplementary file 1 [file ijms-22-03792-s001.zip › Supplementary_File/C_ GO_Analysis_Results/16-30nt_go_Reston-24h-Huh7_vs_Control-24h-Huh7_up.mature_mirna_targets/BP_FoldEnrichmentDotPlot.pdf]

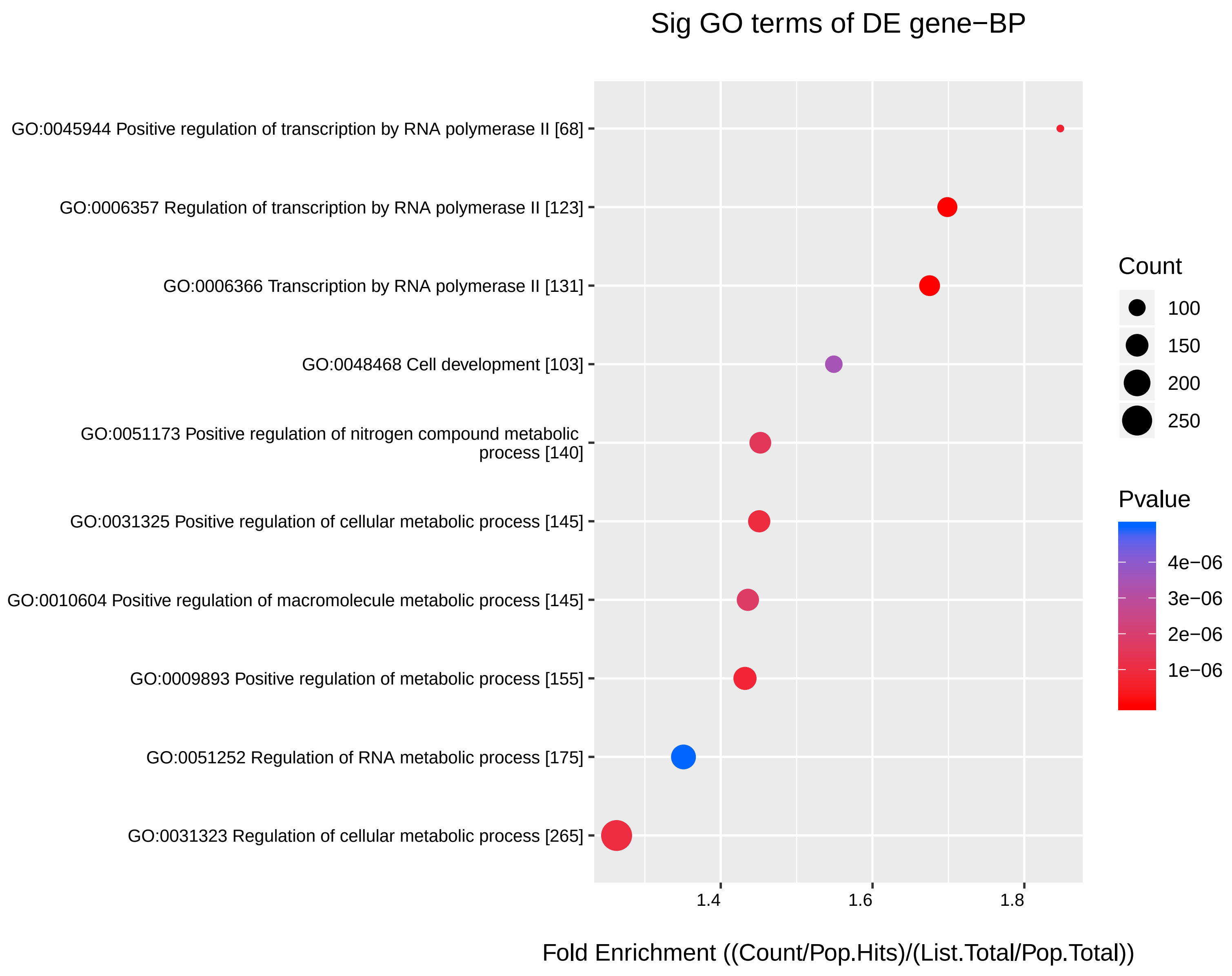

Sig GO terms of DE gene−BP
GO:0045944 Positive regulation of transcription by RNA polymerase II [68]
GO:0006357 Regulation of transcription by RNA polymerase II [123]
GO:0006366 Transcription by RNA polymerase II [131]
GO:0048468 Cell development [103]
GO:0051173 Positive regulation of nitrogen compound metabolic process [140]
GO:0031325 Positive regulation of cellular metabolic process [145]
GO:0010604 Positive regulation of macromolecule metabolic process [145]
GO:0009893 Positive regulation of metabolic process [155]
GO:0051252 Regulation of RNA metabolic process [175]
GO:0031323 Regulation of cellular metabolic process [265]
1.4
1.6
1.8
Fold Enrichment ((Count/Pop.Hits)/(List.Total/Pop.Total))
Count
100
150
200
250
Pvalue
4e−06
3e−06
2e−06
1e−06
